# Supplementary material for: Neural signal analysis with memristor arrays towards high-efficiency brain–machine interfaces
Source: Nat Commun. 2020 Aug 25;11:4234. doi: 10.1038/s41467-020-18105-4 (PMC7447752; doi:10.1038/s41467-020-18105-4)
Supplement: Supplementary file 1 — Supplementary Information [file 41467_2020_18105_MOESM1_ESM.pdf]

Supplementary Information

Neural Signal Analysis with Memristor Arrays Towards High-Efficiency Brain-Machine Interfaces

Zhengwu Liu[1], Jianshi Tang[1,2*], Bin Gao[1,2], Peng Yao[1], Xinyi Li[1], Dingkun Liu[3], Ying Zhou[1], He Qian[1,2] Bo Hong[3*] and Huaqiang Wu[1,2*]

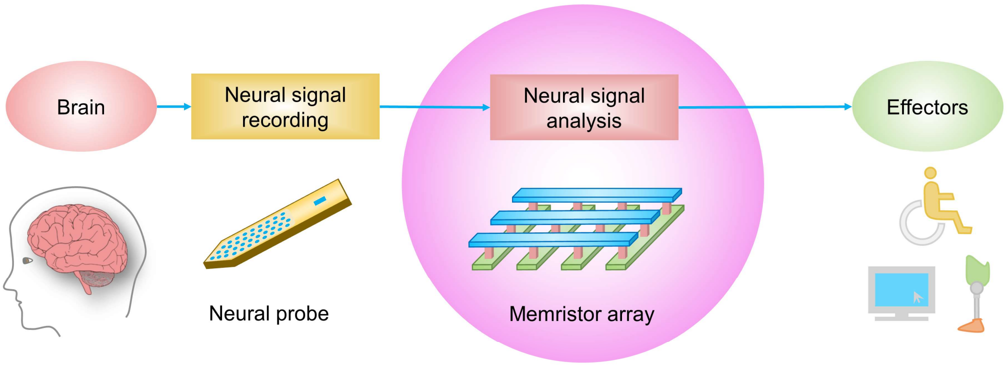

Supplementary Figure 1. Schematic of the memristor-based neural signal analysis system in a complete BMI. The memristor array plays the central role in this BMI. It translates the neural probe-recorded neural signals to control commands for the external effectors, such as a prosthesis or a mouse.

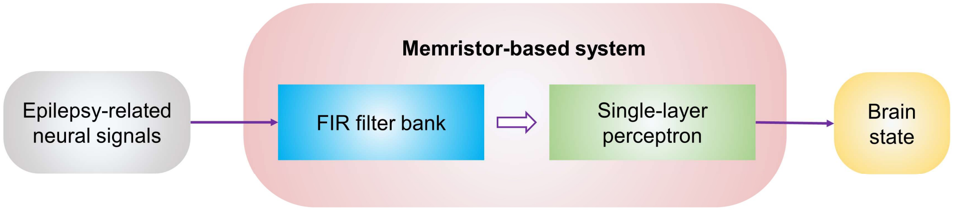

Supplementary Figure 2. Schematic of epilepsy-related neural signal analysis using memristor-based system. The memristor-based system for epilepsy-related neural signal analysis mainly includes an FIR filter bank (as a signal pre-processor) and a single-layer perceptron neural network (as a decoder).

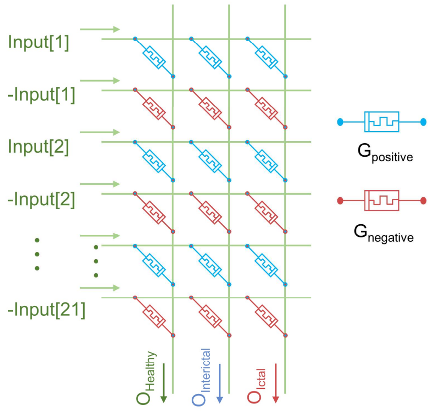

Supplementary Figure 3. Implementation of the neural network. Implementation of the single-layer perceptron neural network in a memristor array to identify the brain states using the filtered waves in 4 different frequency bands.

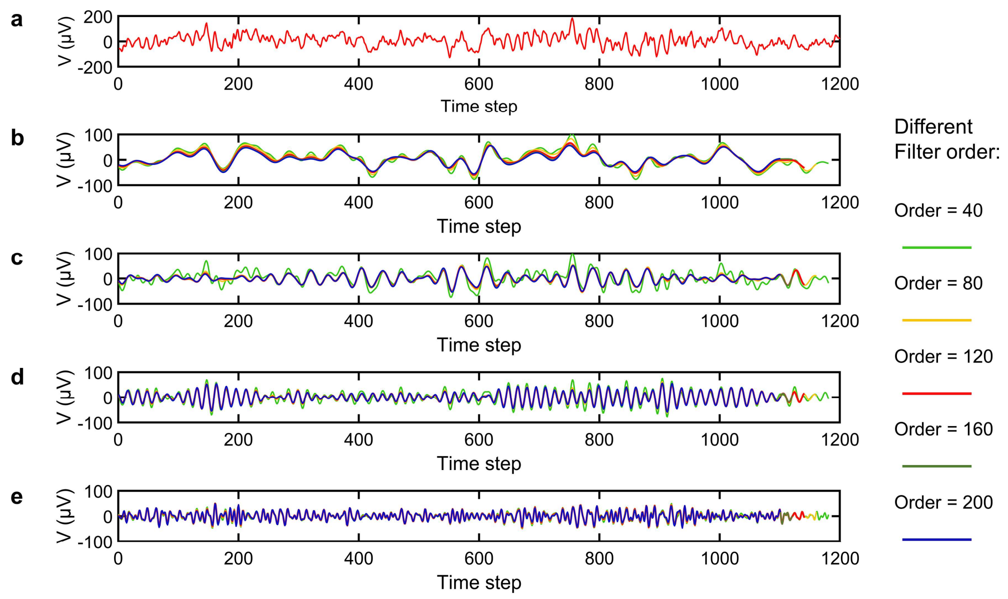

Supplementary Figure 4. Selection of the filter order. a, Test input signal, which is from the Bonn Epilepsy dataset. **b-e,** The filtered waveforms for four filters δ, θ, α and β are shown from top to bottom. In every panel, there are 5 filtered waveforms which are from FIR filter with different orders, 40, 80,120,160 and 200. From theses resulted waveforms, especially for filter θ, orders 120,160 and 200 result in similar waveforms, which are different from results by filters with orders 40 and 80. Thus, we choose the order 120 for our memristor-array-based filter bank.

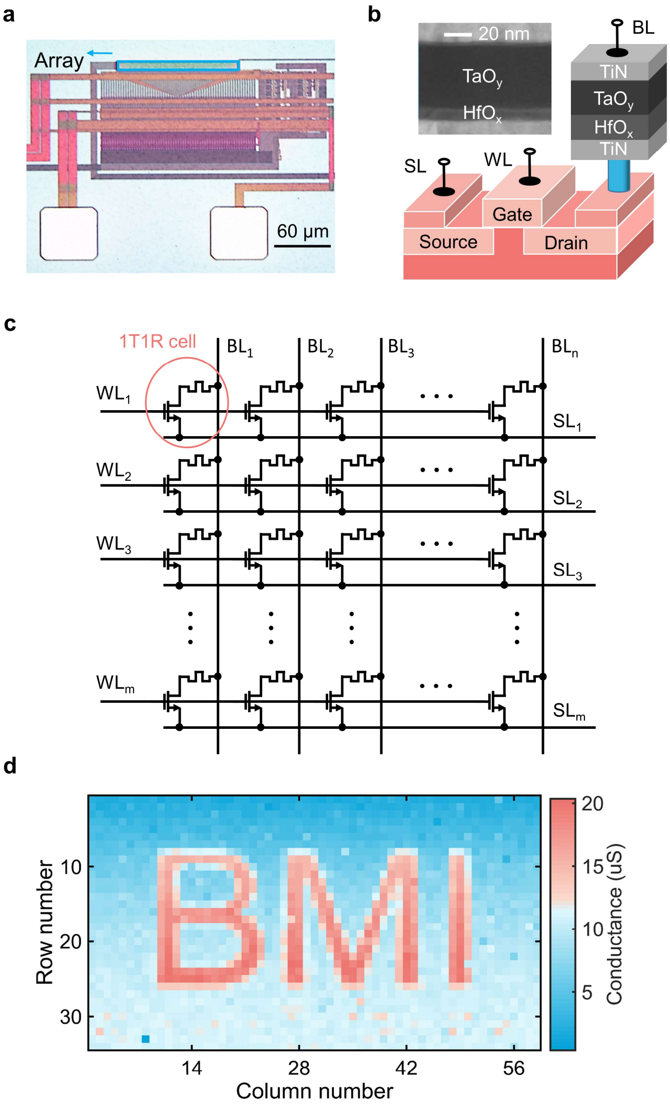

Supplementary Figure 5. Device and array structures of TiN/HfO_x/TaO_y/TiN memristor. a, Microscope image of the 1k-bit 1T1R array used in this work (scale bar, 60 μm). **b,** Schematic illustration of the 1T1R structure with TiN/HfO_x/TaO_y/TiN memristor. The inset is the cross-sectional transmission electron micrograph (TEM) of the memristor material stack. The scale bar in the inset equals 20 nm. **c,** A 1T1R memristor cell has three terminals: transistor gate, transistor source and top electrode. In the array architecture, top electrodes of devices in one column are connected together to a bit line (BL). Transistor gates and sources in one row are connected to a word line (WL) and source line (SL) respectively. **d,** Conductance mapping on the memristor array to display the letter “BMI”.

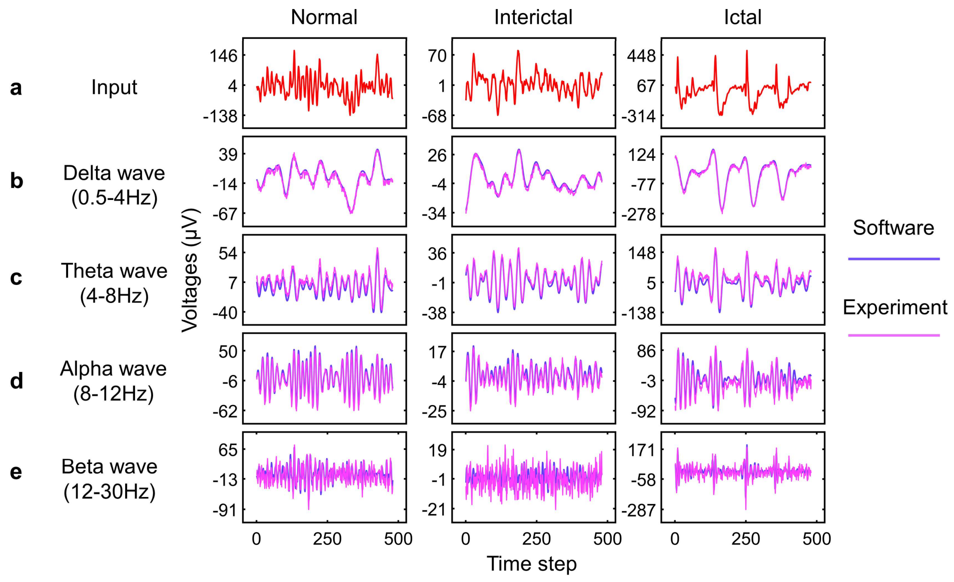

Supplementary Figure 6. Complete filtering results from the filter bank. a, 3 kinds of epilepsy-related raw neural signals are fed as the input for the filter bank. The filtered results of the filters δ, θ, α and β are shown in **b, c, d** and **e**, respectively. The blue line represents software-calculated results and the purple line represents experimental results.

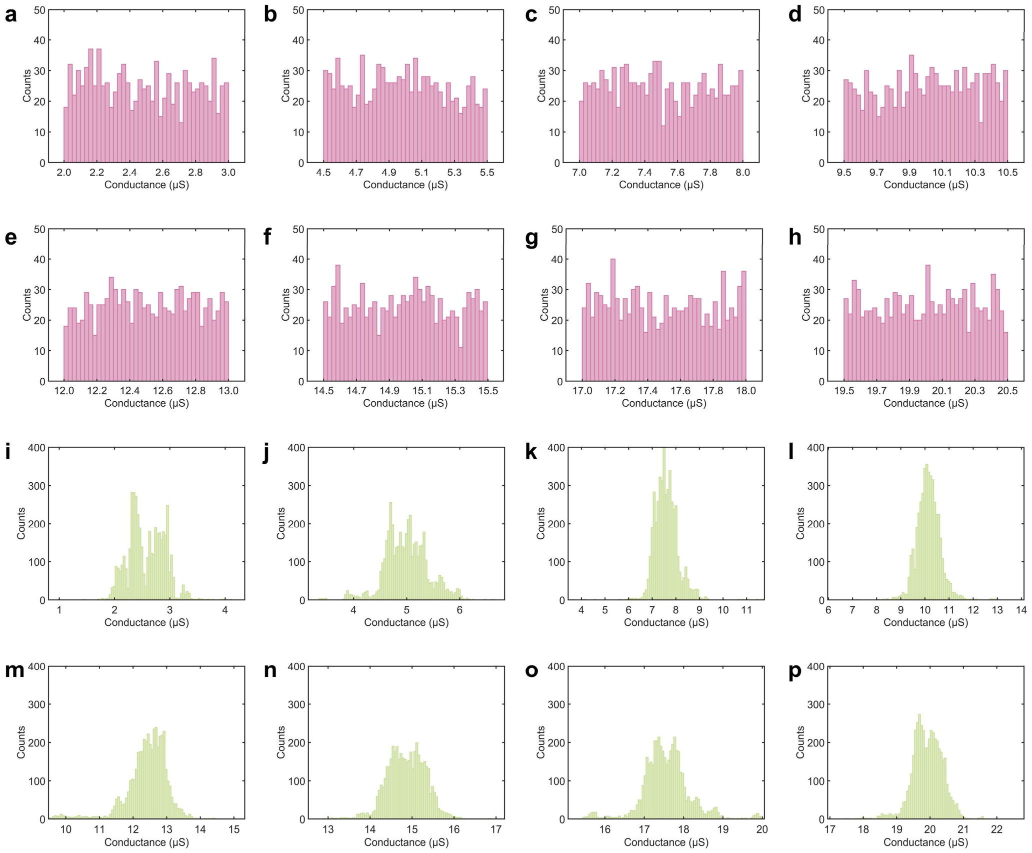

Supplementary Figure 7. Conductance distributions of memristors in the mapping and read operations. a-h, Mapped conductance distributions of eight typical levels: 2.5, 5.0, 7.5, 10.0, 12.5, 15.0, 17.5 and 20 μS. Each distribution includes the mapping results of 1000 memristors. **i-p,** Read noise distributions of these eight conductance levels. Each distribution is obtained from 100-times read results.

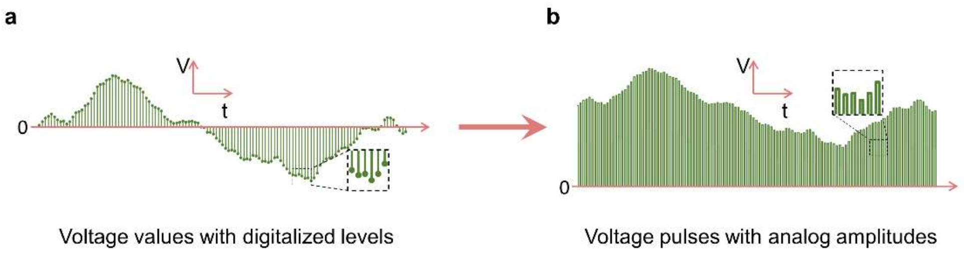

Supplementary Figure 8. Representation of neural signal inputs for memristor-based system. Sampled neural signals with digitalized values (**a**) in the Bonn Epilepsy Dataset are transformed to voltages pulses with analog amplitudes (**b**) and then applied on the memristor array. For example, a 121-point signal clip labeled as the interictal brain state is shown here. The pulse width is 50 ns in this work.
